# Synthesis and structural characterization of hexa-μ_2_-chlorido-μ_4_-oxido-tetra­kis­{[4-(phenyl­ethyn­yl)pyridine-κ*N*]copper(II)} di­chloro­methane monosolvate

**DOI:** 10.1107/S2056989020015935

**Published:** 2021-01-01

**Authors:** Rayya A. Al Balushi, Muhammad S. Khan, Md. Serajul Haque Faizi, Ashanul Haque, Kieran Molloy, Paul R. Raithby

**Affiliations:** a Department of Basic Sciences, College of Applied and Health Science, Al Sharqiyah University, PO Box 42, Ibra 400, Sultanate of Oman; b Department of Chemistry, Sultan Qaboos University, PO Box 36, Al-Khod 123, Sultanate of Oman; c Department of Chemistry, Langat Singh College, B.R.A. Bihar University, Muzaffarpur, Bihar 842001, India; d Department of Chemistry, College of Science, University of Hail, Kingdom of Saudi Arabia; e Department of Chemistry, University of Bath, Claverton Down, Bath BA2 7AY, UK

**Keywords:** crystal structure, 4-phenyl­ethynyl-pyridine, tetra­hedral

## Abstract

In the crystal structure of the title compound, [Cu_4_Cl_6_O(C_13_H_9_N)_4_]·CH_2_Cl_2_, the core mol­ecular structure consists of a Cu_4_ tetra­hedron with a central inter­stitial O atom. Each edge of the Cu_4_ tetra­hedron is bridged by a chlorido ligand. Each copper(II) cation is coordinated to the central O atom, two chlorido ligands and one *N* atom of the 4-phenyl­ethynyl­pyridine ligand. In the crystal, the mol­ecules are linked by inter­molecular C—H⋯Cl inter­actions. Furthermore, C—H⋯π and π–π inter­actions also connect the mol­ecules, forming a three-dimensional network. Hirshfeld surface analysis indicates that the most important contributions for the packing arrangement are from H⋯H and C⋯H/H⋯C inter­actions.

## Abstract 2

The title compound, [Cu_4_Cl_6_O(C_13_H_9_N)_4_], was obtained by the reaction of CuCl with 4-phenyl­ethynyl­pyridine in di­chloro­methane. The complex contains a tetra­hedron of four Cu^II^ cations coordinated to a central μ_4_-O atom, with the six edges of the Cu_4_ tetra­hedron bridged by Cl atoms. The Cu—O distances average 1.905 Å and Cu—Cl 2.418 Å.

## Chemical context   

Polynuclear Cu^II^ complexes with various bridges between the metal centres have attracted much attention in the past decade, from both an experimental and a theoretical point of view, and a significant amount of research has been devoted to analysing their structural and magnetic properties (Bertrand & Kelley, 1966 ▸). Copper complexes that form clusters of the type Cu_4_O*X*_6_ *L*_4_ (*X* = halogen, *L* = ligand or *X*) are known (Bertrand *et al.*, 1968 ▸; Dey *et al.*, 2002 ▸; Mukherjee *et al.*, 2007 ▸; Thakurta *et al.*, 2009 ▸; Wegner *et al.*, 2001 ▸). In our studies on dimeric, tetra­meric, and polymeric Cu complexes supported by ethynyl­pyridine-based ligands, we have obtained Cu_4_O*X*_6_ *L*_4_ complexes where a central oxide ion is tetra­hedrally coord­inated to four copper ions, which are in turn bridged in pairs by six chloride ions, and the *L* groups complete the trigonal–bipyramidal coordination of the copper centres. The structural complexity of these [Cu_4_O*X*_6_ *L*_4_] systems, as well as their challenging magnetic properties, has promoted sustained structural work on the subject (Atria *et al.*, 1999 ▸), where the magnetic properties exhibited by the compound were successfully modelled in a rather simple and elegant fashion. We report herein the synthesis of the title complex μ_4_-oxo-hexa-μ_2_-chlorido-tetra­kis­[(4-phenyl­ethynyl­pyridine)­copper(II)] di­chloro­methane solvate (**1**) from 4-(2-phenyl­ethyn­yl)pyridine and CuCl in di­chloro­methane. It is well known that Cu*X* (*X* = Cl, Br, I) salts react with ethynyl­pyridine-based ligands in di­chloro­methane to form coordination-driven self-assembled tetra­hedral Cu^I^ complexes; however, oxidation to form Cu^II^ species is also possible. We have a long-standing inter­est in the design and development of functional ethynyl-based carbocyclic and heterocyclic ligands and their transition metal complexes (Haque *et al.*, 2018 ▸; Haque *et al.*, 2019*a* ▸). In the past, we have reported several dimeric, tetra­meric, and polymeric Cu^I^ complexes supported by ethynyl­pyridine-based ligands. (Al-Balushi *et al.*, 2016*a* ▸,*b* ▸; Ilmi *et al.*, 2018 ▸). In the quest for new dimeric halide-bridged Cu complexes, we obtained an oxidized Cu^II^ product, compound **1**. Our experience suggests that the chloride-containing Cu^I^ complexes are somewhat less stable and oxidize easily (*in situ* or during crystallization), leading to the formation of multiple products. The crystal structure, as well as Hirshfeld surface analysis, indicate that the most important contributions to the packing arrangement within are from H⋯H and C⋯H/H⋯C inter­actions.
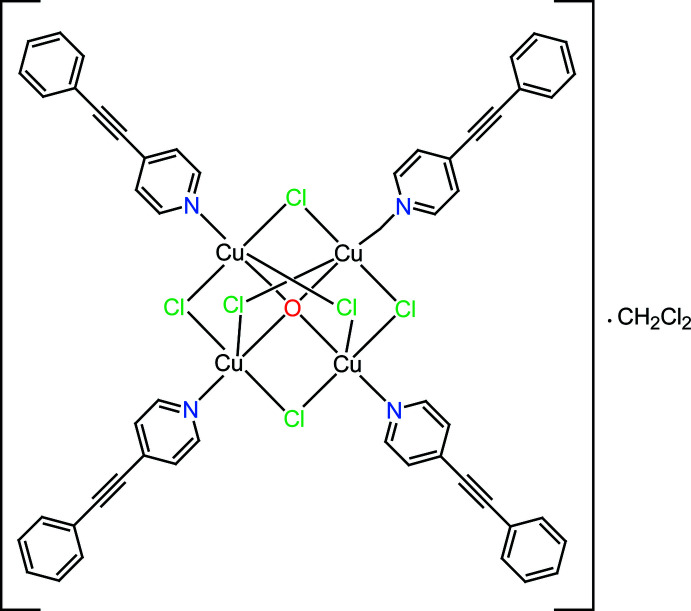


## Structural commentary   

**Figure 1:**
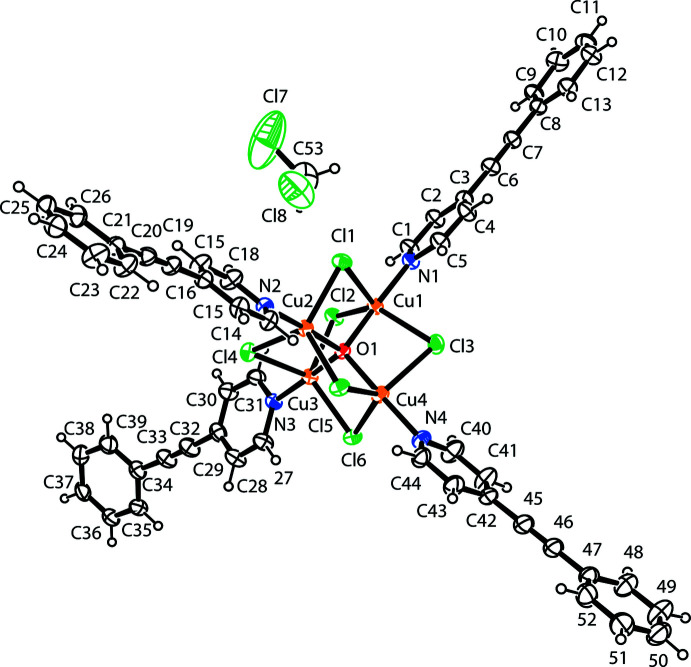
The molecular structure of the title compound with atom labelling and displacement ellipsoids drawn at the 40% probability level.

Within the crystal structure the asymmetric unit consists of a central core with an O atom binding four Cu atoms, and there are six bridging Cl atoms, with four 4-phenyl­ethynyl­pyridine units also attached to the Cu atoms *via* the N atoms of the six-membered heterocyclic ring (Fig. 1 ▸). The Cu atoms are tetra­hedrally oriented about the O atom and are bridged by the six Cl atoms, which adopt an octa­hedral arrangement with respect to the cluster centre. Each Cu atom lies at the centre of a trigonal bipyramid, with the O atom and the 4-phenyl­ethynyl-pyridine N atom in the axial positions and three Cl atoms in the equatorial positions. Average distances are: Cu—N, 1.976 (3) Å; Cu—O, 1.905 (2) Å; Cu—Cl1, 2.418 (11) Å; Cu—Cu, 3.111 (2) Å. The average of the axial O—Cu—N angles is 177.1 (12)°; that of the equatorial C1—Cu—C1 angles is 119.2 (4)°. The dihedral angles between benzene and pyridine rings are 0.9 (2)° (C21–C26 and C14–C18/N2), 34.0 (3)° (C34–C39 and C27–C31/N3), 5.8 (3)° (C47–C52 and C40–C44/N4) and 5.7 (3)° (C8–C13 and C1–C5/N1). The average distance of the triple bond is 1.193 (6) Å;.

## Supra­molecular features   

**Figure 2:**
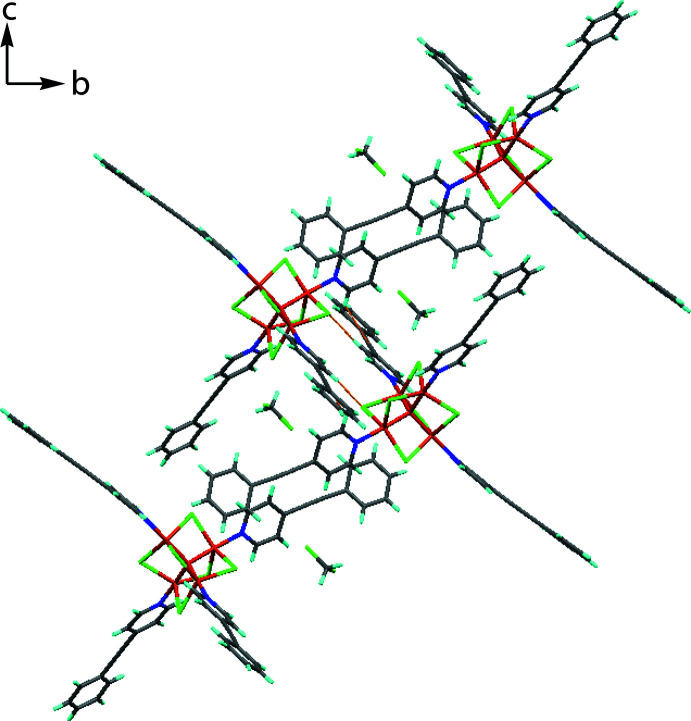
Crystal packing of the title compound showing the C—H⋯Cl inter­actions.

**Figure 3:**
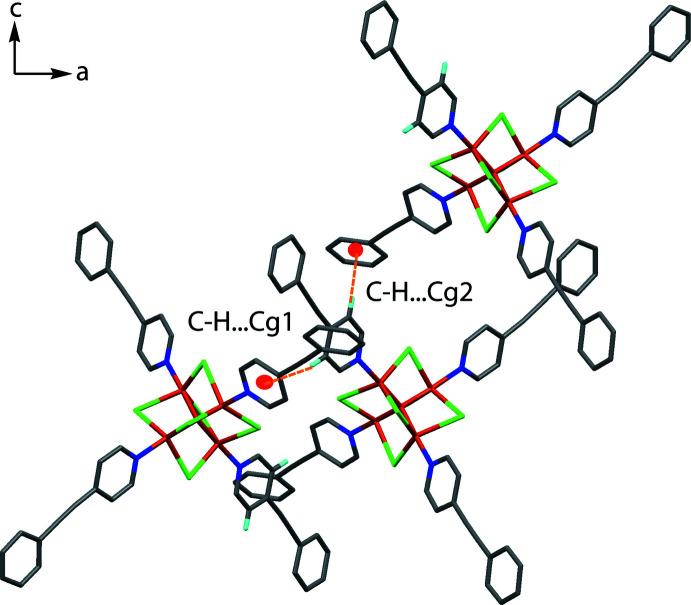
Crystal packing of the title compound showing the C41—H41⋯*Cg*1 and C43—H43⋯*Cg*2 inter­actions viewed along the *b*-axis direction.

The crystal structure of the title compound is consolidated by several inter- and intra­molecular inter­actions, the presence of which are supported by a Hirshfeld surface analysis. In the crystal, the presence of several C—H⋯Cl (Fig. 2 ▸, Table 1 ▸) inter­actions (C1—H1⋯Cl2, C4—H4⋯Cl5^i^, C14—H14⋯Cl5, C17—H17⋯Cl4^ii^, C27—H27⋯Cl6, C31—H31⋯Cl2, C44—H44⋯Cl5, C53—H53*A*⋯Cl1 and C53—H53*B*⋯Cl3^i^ helps in the stabilization of the crystals. Furthermore, C31—H31⋯π (π is the midpoint of the C19≡C20 triple bond) inter­actions connect the mol­ecules into a chain along the *b-*axis direction. The C41—H41⋯*Cg*1 and C43—H43⋯*Cg*2 inter­actions form a network along the *b-*axis direction (Fig. 3 ▸). π–π inter­actions [centroid⋯centroid = 3.672 (3) Å; between the C47–C52 and N4/C40–C44 are also present and are supported by the Hirshfeld surface analysis.

## Hirshfeld surface analysis   

**Figure 4:**
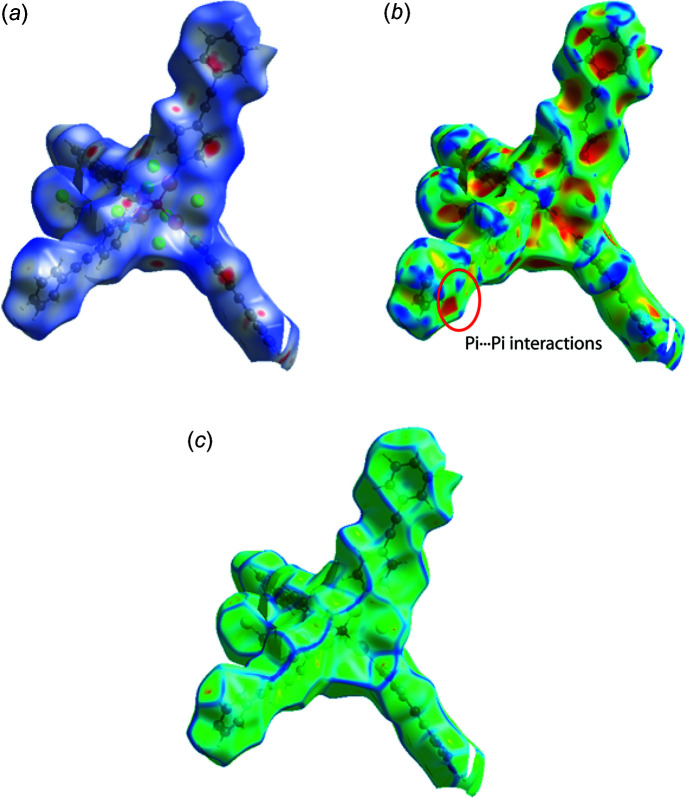
Hirshfeld surfaces of the title mol­ecule plotted over (*a*) *d*_norm_ (*b*) shape-index showing the π–π stacking and (*c*) curvedness.

**Figure 5:**
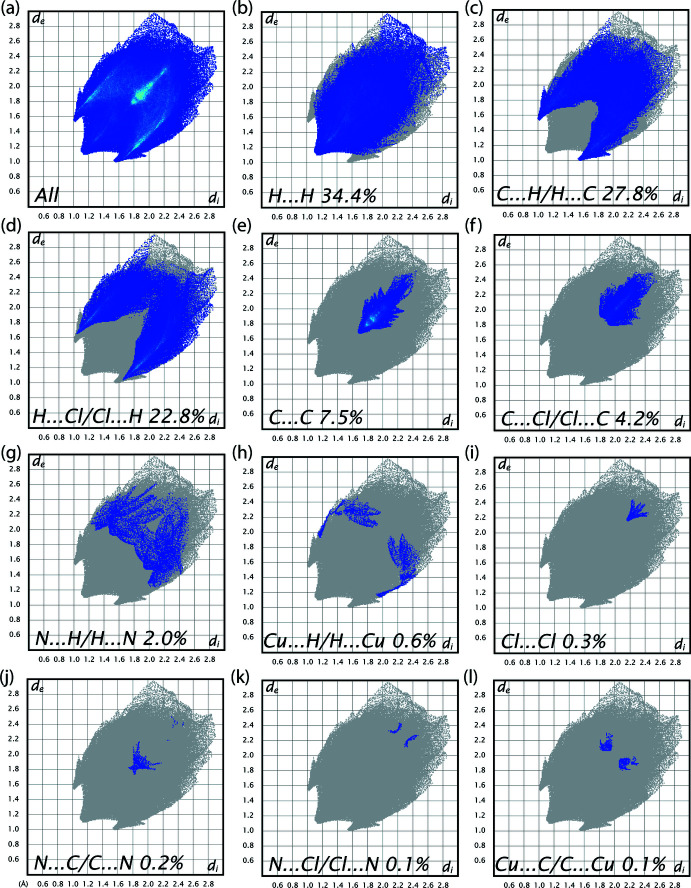
A view of the two-dimensional fingerprint plots for the title compound, showing (*a*) all inter­actions, and delineated into (*b*) H⋯H, (*c*) C⋯H/H⋯C, (*d*) Cl⋯H/H⋯Cl, (*e*) C⋯C and (*f*) C⋯Cl/Cl⋯C (*g*) N⋯H/H⋯N (*h*) Cu⋯H/H⋯Cu (i) Cl⋯Cl (*j*) N⋯C/C⋯N (*k*) N⋯Cl/Cl⋯N and (*l*) Cu⋯C/C⋯Cu inter­actions

In order to better visualize and analyse the role of weak inter­molecular contacts in the crystal, a Hirshfeld surface (HS) analysis (Spackman & Jayatilaka, 2009 ▸) was carried out and the associated two-dimensional fingerprint plots (McKinnon *et al.*, 2007 ▸) generated using *CrystalExplorer17.5* (Turner *et al.*, 2017 ▸). The white surface indicates contacts with distances equal to the sum of van der Waals radii, and the red and blue colours indicate distances shorter (in close contact) or longer (distant contact) than the sum of the van der Waals radii, respectively (Venkatesan *et al.*, 2016 ▸). The dark-red spots on the *d*_norm_ surface arise as a result of short inter­atomic contacts (Fig. 4 ▸), while the other weaker inter­molecular inter­actions appear as light-red spots. The red points, which represent close contacts and negative *d*_norm_ values on the surface, correspond to the C—H⋯Cl inter­actions. The shape-index of the Hirshfeld surface is a tool for visualizing the π–π stacking by the presence of adjacent red and blue triangles; if these triangles do not appear, then there are no π–π inter­actions. The plot of the Hirshfeld surface mapped over shape-index shown in Fig. 4 ▸ *b* clearly suggests that there are π–π inter­actions in the crystal packing of the title compound. The curvedness plot (Fig. 4 ▸ *c*) shows flat surface patches characteristic of planar stacking. The large green regions represent a relatively flat (*i.e.* planar) surface area, while the blue regions demonstrate areas of curvature. The presence of π–π stacking interactions is also evident as flat regions on the Hirshfeld surface plotted over curvedness. The percentage contributions of various contacts to the total Hirshfeld surface are shown in the two-dimensional fingerprint plots in Fig. 5 ▸. These indicate that the crystal packing is dominated by H⋯H contacts, representing van der Waals inter­actions (34.4% contribution to the overall surface), followed by C⋯H/H⋯C, C⋯C, Cl⋯H/H⋯Cl, C⋯Cl/Cl⋯C, and N⋯H/H⋯N inter­actions, which contribute 27.8%, 22.8%, 7.5%, 4.2%, and 2.0%, respectively. The other inter­actions (Cu⋯H/H⋯Cu, Cl⋯Cl, N⋯C/C⋯N, N⋯Cl/Cl⋯N and Cu⋯C/C⋯Cu) contribute less than 2% and are not considered to be significant.

## Database survey   

A search of the Cambridge Structural Database (CSD, version 5.39; Groom *et al.*, 2016 ▸) gave ten hits for the Cu_4_O*X*_6_ *L*_4_ moiety. The eight most closely related compounds are hexa-μ_2_-chlorido-tetra­kis­(2-ethyl­pyrazine-*N*)-μ_4_-oxo-tetra­copper(II) (Näther & Jess 2002 ▸), [Cu_4_Cl_6_O(C_6_H_8_N_2_)_4_], in which the Cu_4_ tetra­hedra are centred by an inter­stitial O atom. Each edge of the Cu_4_ tetra­hedron is bridged by a chlorido ligand. The copper(II) cations are fourfold coordinated by one O atom, two chlorido ligands and one N atom of the 2-ethyl­pyrazine ligand within a distorted tetra­hedron. The Cu_4_Cl_6_O(C_6_H_8_N_2_)_4_ units are located in general positions. Three oxo complexes with a tetra­nuclear [Cu_4_(μ-Cl)_6_(μ-O)] unit (Cortés *et al.*, 2006 ▸), namely 4-phenyl-1*H*-imidazolium hexa-μ_2_-chlorido-chlorido-μ_4_-oxo-tris­(4-phenyl-1*H*-imidazole-κ*N* ^1^)tetra­copper(II) monohydrate, (C_9_H_9_N_2_)[Cu_4_Cl_7_O(C_9_H_8_N_2_)_3_]·H_2_O, hexa-μ_2_-chlorido-μ_4_-oxo-tetra­kis­(pyridine *N*-oxide-κ*O*)tetra­copper(II), [Cu_4_Cl_6_O(C_5_H_5_NO)_4_], and hexa-μ_2_-chlorido-tetra­kis­(2-methyl-1*H*-imidazole-κ*N* ^1^)μ_4_-oxo-tetra­copper(II) methanol tris­olvate, [Cu_4_Cl_6_O(C_4_H_6_N_2_)_4_]·3CH_4_O, exhibit the same Cu_4_OCl_6_ framework, where the O atom at the centre of an almost regular tetra­hedron bridges four copper cations at the corners. This group is in turn surrounded by a Cl_6_ octa­hedron, leading to a rather globular species.

## Synthesis and crystallization   

The ligand *L* was prepared by adapting a previously reported procedure (Haque *et al.*, 2019*b* ▸). 1-Ethynyl­benzene (0.33 g, 3.23 mmol) and 4-iodo­pyridine (0.66 g, 3.23 mmol) were dissolved in a ^*i*^Pr_2_NH/THF mixture (1:2, 60 mL) under an argon atmosphere. Catalytic amounts of Pd(OAc)_2_ (3 mg), CuI (3 mg), and PPh_3_ (10 mg) were added to the mixture and it was refluxed overnight. The solvent was then removed under vacuum and the residue was dissolved in di­chloro­methane (100 mL), washed with water and extracted with di­chloro­methane. The combined organic layers were washed with water and brine and then dried over anhydrous magnesium sulfate. The solution was concentrated under vacuum, and the crude product was chromatographed on a silica column using a mixture of hexa­ne:di­chloro­methane (1:1, *v*/*v*). The ligand was obtained as an orange/pale-brown powder (0.51g, 88% yield). IR (ν_max_) cm^−1^: 2185 (–C≡C–), 1590 (C—N). ^1^H NMR (700 MHz, CDCl_3_): δ(ppm) 8.07 (*d*, 2H, *J* = 6.0, H-py), 7.94 (*d*, 2H, *J* = 6.2, H-py), 7.73 (*d*, 2H, *J* = 6.2, H-ph), 6.80–6.74 (*m*, 3H, H-ph). ESI–MS: *m*/*z* 179.06 (*M*+). C_13_H_9_N Analysis calculated: C, 87.12; H 5.06; N, 7.82%. Found: C, 86.65; H, 4.89; N, 7.67%.

**Synthesis of Cu_4_OCl_6_** ***L*** **_4_ [** ***L*** **= 4-(2-phenyl­ethyn­yl)pyridine] (1)**

The title complex **1** was obtained by the reaction of the ethynyl­pyridine-based ligand with Cu^I^Cl due to partial oxidation under the reaction conditions employed. The methodology for the synthesis of the complex is as follows: *L* (0.050 g, 0.24 mmol) and CuCl (0.024 g, 0.24 mmol) were dissolved in di­chloro­methane (50 mL). The reaction mixture was stirred at room temperature under a partial argon atmosphere for 24 h, after which period the solvent was removed under reduced pressure. The crude product was dissolved in di­chloro­methane and filtered through a pad of celite using di­chloro­methane giving the final product as an orange powder (0.057 g, 79% yield). Diffusion of hexane to a di­chloro­methane solution gave the final product as orange crystals.

## Refinement   

Crystal data, data collection and structure refinement details are summarized in Table 2 ▸. H atoms were positioned with idealized geometry (C—H = 0.95–0.99 Å) and refined with fixed isotropic displacement parameters [*U*_iso_(H) = 1.2*U*_eq_(C)] using a riding model.

## Supplementary Material

Crystal structure: contains datablock(s) I. DOI: 10.1107/S2056989020015935/mw2170sup1.cif

Structure factors: contains datablock(s) I. DOI: 10.1107/S2056989020015935/mw2170Isup2.hkl

CCDC reference: 2048458

Additional supporting information:  crystallographic information; 3D view; checkCIF report

## Figures and Tables

**Table 1:** Hydrogen-bond geometry (Å, °) π is the midpoint of the C19≡C20 triple bond. *Cg*1 and *Cg*2 are the centroids of the N3/C27–C31 and C34–C39 rings, respectively.

*D*—H⋯*A*	*D*—H	H⋯*A*	*D*⋯*A*	*D*—H⋯*A*
C1—H1⋯Cl2	0.95	2.64	3.283 (4)	126
C4—H4⋯Cl5^i^	0.95	2.90	3.697 (4)	142
C14—H14⋯Cl5	0.95	2.52	3.211 (4)	130
C17—H17⋯Cl4^ii^	0.95	2.80	3.609 (5)	144
C27—H27⋯Cl6	0.95	2.74	3.297 (5)	118
C31—H31⋯Cl2	0.95	2.71	3.345 (4)	125
C44—H44⋯Cl5	0.95	2.61	3.257 (5)	126
C53—H53*A*⋯Cl1	0.99	2.97	3.808 (9)	143
C53—H53*B*⋯Cl3^i^	0.99	2.79	3.771 (8)	172
C31—H31⋯π	0.95	2.84	3.600 (3)	135
C41—H41⋯*Cg*1^iii^	0.95	2.78	3.705 (6)	165
C43—H43⋯*Cg*2	0.95	2.73	3.452 (2)	150

Symmetry codes: (i) 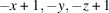; (ii) 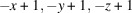; (iii) 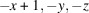.

**Table 2:** Experimental details

Crystal data	
Chemical formula	[Cu_4_Cl_6_O(C_13_H_9_N)_4_]·CH_2_Cl_2_
*M*_r_	1284.63
Crystal system, space group	Triclinic, *P* 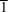
Temperature (K)	150
*a*, *b*, *c* (Å)	12.7166 (2), 14.4366 (2), 16.4038 (3)
α, β, γ (°)	105.024 (1), 105.935 (1), 102.999 (1)
*V* (Å^3^)	2650.81 (8)
*Z*	2
Radiation type	Mo *K*α
μ (mm^−1^)	2.03
Crystal size (mm)	0.15 × 0.12 × 0.12

Data collection	
Diffractometer	Nonius Kappa CCD
Absorption correction	Multi-scan (*SORTAV*; Blessing 1995 ▸)
*T*_min_, *T*_max_	0.548, 0.572
No. of measured, independent and observed [*I* > 2σ(*I*)] reflections	50117, 12164, 8495
*R*_int_	0.060
(sin θ/λ)_max_ (Å^−1^)	0.651

Refinement	
*R*[*F* ^2^ > 2σ(*F* ^2^)], *wR*(*F* ^2^), *S*	0.049, 0.127, 1.02
No. of reflections	12164
No. of parameters	631
H-atom treatment	H-atom parameters constrained
Δρ_max_, Δρ_min_ (e Å^−3^)	0.89, −0.99

Computer programs: *COLLECT* (Nonius, 1998 ▸), *HKL* and *SCALEPACK* (Otwinowski & Minor, 1997 ▸), *SHELXS97* (Sheldrick, 2008 ▸), *SHELXL2017* (Sheldrick, 2015 ▸), *ORTEPIII* (Burnett & Johnson, 1996 ▸), *ORTEP-3 for Windows* (Farrugia, 2012 ▸) and *publCIF* (Westrip, 2010 ▸).

## supplementary crystallographic information

### Crystal data

**Table:**

[Cu_4_Cl_6_O(C_13_H_9_N)_4_]·CH_2_Cl_2_	*Z* = 2
*M**_r_* = 1284.63	*F*(000) = 1288
Triclinic, *P*1	*D*_x_ = 1.609 Mg m^−^^3^
*a* = 12.7166 (2) Å	Mo *K*α radiation, λ = 0.71073 Å
*b* = 14.4366 (2) Å	Cell parameters from 38646 reflections
*c* = 16.4038 (3) Å	θ = 2.9–27.5°
α = 105.024 (1)°	µ = 2.03 mm^−^^1^
β = 105.935 (1)°	*T* = 150 K
γ = 102.999 (1)°	Block, brown
*V* = 2650.81 (8) Å^3^	0.15 × 0.12 × 0.12 mm

### Data collection

**Table:**

Nonius Kappa CCD diffractometer	12164 independent reflections
Radiation source: fine-focus sealed tube	8495 reflections with *I* > 2σ(*I*)
Graphite monochromator	*R*_int_ = 0.060
274 2.0 degree images with φ and ω scans	θ_max_ = 27.6°, θ_min_ = 3.0°
Absorption correction: multi-scan (Sortav; Blessing 1995)	*h* = −16→16
*T*_min_ = 0.548, *T*_max_ = 0.572	*k* = −18→18
50117 measured reflections	*l* = −21→21

### Refinement

**Table:**

Refinement on *F*^2^	0 restraints
Least-squares matrix: full	Hydrogen site location: inferred from neighbouring sites
*R*[*F*^2^ > 2σ(*F*^2^)] = 0.049	H-atom parameters constrained
*wR*(*F*^2^) = 0.127	*w* = 1/[σ^2^(*F*_o_^2^) + (0.0564*P*)^2^ + 3.7541*P*] where *P* = (*F*_o_^2^ + 2*F*_c_^2^)/3
*S* = 1.02	(Δ/σ)_max_ = 0.001
12164 reflections	Δρ_max_ = 0.89 e Å^−^^3^
631 parameters	Δρ_min_ = −0.99 e Å^−^^3^

### Special details

**Table:**

Geometry. All esds (except the esd in the dihedral angle between two l.s. planes) are estimated using the full covariance matrix. The cell esds are taken into account individually in the estimation of esds in distances, angles and torsion angles; correlations between esds in cell parameters are only used when they are defined by crystal symmetry. An approximate (isotropic) treatment of cell esds is used for estimating esds involving l.s. planes.

### Fractional atomic coordinates and isotropic or equivalent isotropic displacement parameters (Å^2^)

**Table:**

	*x*	*y*	*z*	*U*_iso_*/*U*_eq_	
Cu1	0.59753 (4)	0.13110 (3)	0.37484 (3)	0.02947 (12)	
Cu2	0.39563 (4)	0.22081 (3)	0.36568 (3)	0.02945 (12)	
Cu3	0.53201 (4)	0.23908 (4)	0.23694 (3)	0.02972 (12)	
Cu4	0.37106 (4)	0.02979 (4)	0.20606 (3)	0.03326 (13)	
Cl1	0.51186 (8)	0.19294 (8)	0.49034 (6)	0.0369 (2)	
Cl2	0.71949 (7)	0.25680 (8)	0.34475 (7)	0.0365 (2)	
Cl3	0.49178 (8)	−0.04512 (7)	0.29070 (7)	0.0408 (2)	
Cl4	0.45580 (8)	0.35909 (7)	0.31625 (7)	0.0344 (2)	
Cl5	0.22503 (7)	0.07846 (8)	0.25227 (6)	0.0355 (2)	
Cl6	0.43774 (8)	0.09701 (8)	0.10345 (6)	0.0367 (2)	
Cl7	0.4867 (3)	0.3790 (3)	0.7675 (4)	0.240 (2)	
Cl8	0.2612 (2)	0.2324 (2)	0.6751 (2)	0.1420 (10)	
O	0.47340 (19)	0.15499 (18)	0.29649 (16)	0.0258 (5)	
N1	0.7198 (3)	0.0992 (2)	0.4559 (2)	0.0326 (7)	
N2	0.3071 (3)	0.2858 (2)	0.4319 (2)	0.0321 (7)	
N3	0.5893 (3)	0.3317 (3)	0.1777 (2)	0.0321 (7)	
N4	0.2701 (3)	−0.1021 (3)	0.1125 (2)	0.0385 (8)	
C1	0.8321 (3)	0.1366 (3)	0.4721 (3)	0.0387 (10)	
H1	0.855892	0.183883	0.444621	0.046*	
C2	0.9156 (3)	0.1107 (3)	0.5265 (3)	0.0386 (10)	
H2	0.994667	0.139724	0.535835	0.046*	
C3	0.8834 (3)	0.0419 (3)	0.5673 (3)	0.0353 (9)	
C4	0.7655 (3)	0.0014 (3)	0.5498 (3)	0.0404 (10)	
H4	0.739232	−0.046548	0.575925	0.048*	
C5	0.6884 (3)	0.0315 (3)	0.4949 (3)	0.0411 (10)	
H5	0.608589	0.003118	0.483687	0.049*	
C6	0.9675 (3)	0.0144 (3)	0.6270 (3)	0.0386 (10)	
C7	1.0352 (3)	−0.0082 (3)	0.6781 (3)	0.0365 (9)	
C8	1.1148 (3)	−0.0365 (3)	0.7408 (3)	0.0364 (9)	
C9	1.2335 (3)	0.0069 (3)	0.7646 (3)	0.0424 (10)	
H9	1.262150	0.052823	0.737537	0.051*	
C10	1.3093 (4)	−0.0173 (4)	0.8278 (3)	0.0504 (12)	
H10	1.390090	0.013447	0.845156	0.060*	
C11	1.2687 (4)	−0.0852 (4)	0.8653 (3)	0.0490 (11)	
H11	1.321158	−0.102063	0.908199	0.059*	
C12	1.1512 (4)	−0.1293 (4)	0.8409 (3)	0.0497 (11)	
H12	1.123184	−0.176620	0.866944	0.060*	
C13	1.0749 (4)	−0.1053 (3)	0.7794 (3)	0.0436 (10)	
H13	0.994295	−0.136030	0.763053	0.052*	
C14	0.1961 (3)	0.2383 (3)	0.4150 (3)	0.0375 (9)	
H14	0.161600	0.171347	0.372599	0.045*	
C15	0.1298 (3)	0.2814 (3)	0.4557 (3)	0.0382 (9)	
H15	0.051425	0.244242	0.441431	0.046*	
C16	0.1764 (3)	0.3785 (3)	0.5174 (3)	0.0346 (9)	
C17	0.2923 (4)	0.4281 (4)	0.5358 (3)	0.0470 (11)	
H17	0.328895	0.494974	0.578058	0.056*	
C18	0.3531 (4)	0.3793 (3)	0.4920 (3)	0.0445 (11)	
H18	0.432019	0.414203	0.505436	0.053*	
C19	0.1059 (4)	0.4239 (3)	0.5592 (3)	0.0372 (9)	
C20	0.0397 (4)	0.4535 (3)	0.5895 (3)	0.0380 (9)	
C21	−0.0431 (4)	0.4866 (3)	0.6243 (3)	0.0364 (9)	
C22	−0.1552 (4)	0.4217 (4)	0.5946 (3)	0.0485 (11)	
H22	−0.176695	0.355923	0.551746	0.058*	
C23	−0.2351 (4)	0.4526 (4)	0.6270 (3)	0.0547 (13)	
H23	−0.311833	0.408101	0.606374	0.066*	
C24	−0.2043 (4)	0.5475 (4)	0.6892 (3)	0.0477 (11)	
H24	−0.259810	0.568142	0.711595	0.057*	
C25	−0.0936 (4)	0.6128 (4)	0.7195 (3)	0.0467 (11)	
H25	−0.073483	0.678680	0.761853	0.056*	
C26	−0.0106 (4)	0.5826 (3)	0.6880 (3)	0.0424 (10)	
H26	0.066445	0.626733	0.709696	0.051*	
C27	0.5272 (4)	0.3257 (4)	0.0946 (3)	0.0436 (10)	
H27	0.458081	0.270477	0.061050	0.052*	
C28	0.5593 (4)	0.3960 (4)	0.0562 (3)	0.0483 (11)	
H28	0.512469	0.389894	−0.002329	0.058*	
C29	0.6621 (4)	0.4772 (3)	0.1043 (3)	0.0415 (10)	
C30	0.7252 (4)	0.4840 (3)	0.1907 (3)	0.0430 (10)	
H30	0.793922	0.539021	0.226412	0.052*	
C31	0.6865 (4)	0.4095 (3)	0.2239 (3)	0.0400 (10)	
H31	0.731096	0.413844	0.282489	0.048*	
C32	0.6997 (4)	0.5538 (4)	0.0677 (3)	0.0498 (12)	
C33	0.7328 (4)	0.6179 (4)	0.0394 (3)	0.0486 (11)	
C34	0.7732 (4)	0.6993 (4)	0.0096 (3)	0.0443 (10)	
C35	0.7567 (4)	0.6839 (4)	−0.0809 (3)	0.0432 (10)	
H35	0.717866	0.617900	−0.124341	0.052*	
C36	0.7962 (4)	0.7634 (4)	−0.1082 (3)	0.0435 (10)	
H36	0.785363	0.752164	−0.170008	0.052*	
C37	0.8509 (4)	0.8585 (4)	−0.0459 (3)	0.0506 (12)	
H37	0.877916	0.913099	−0.064975	0.061*	
C38	0.8675 (5)	0.8766 (4)	0.0439 (4)	0.0662 (15)	
H38	0.905580	0.943093	0.086590	0.079*	
C39	0.8278 (5)	0.7963 (4)	0.0718 (3)	0.0577 (13)	
H39	0.838400	0.808259	0.133693	0.069*	
C40	0.3174 (4)	−0.1621 (4)	0.0713 (3)	0.0555 (13)	
H40	0.397743	−0.137270	0.082813	0.067*	
C41	0.2561 (4)	−0.2598 (4)	0.0119 (4)	0.0600 (14)	
H41	0.293855	−0.299850	−0.017452	0.072*	
C42	0.1388 (4)	−0.2987 (3)	−0.0044 (3)	0.0419 (10)	
C43	0.0893 (4)	−0.2333 (3)	0.0350 (3)	0.0483 (11)	
H43	0.008513	−0.254493	0.022820	0.058*	
C44	0.1567 (4)	−0.1368 (3)	0.0923 (3)	0.0464 (11)	
H44	0.120298	−0.092934	0.118602	0.056*	
C45	0.0715 (4)	−0.4002 (4)	−0.0610 (3)	0.0470 (11)	
C46	0.0100 (4)	−0.4851 (3)	−0.1066 (3)	0.0468 (11)	
C47	−0.0649 (4)	−0.5861 (3)	−0.1609 (3)	0.0466 (11)	
C48	−0.0220 (5)	−0.6593 (4)	−0.1977 (3)	0.0590 (13)	
H48	0.058499	−0.642711	−0.186536	0.071*	
C49	−0.0926 (5)	−0.7556 (4)	−0.2500 (4)	0.0711 (16)	
H49	−0.060809	−0.805330	−0.273686	0.085*	
C50	−0.2091 (5)	−0.7803 (4)	−0.2681 (4)	0.0692 (16)	
H50	−0.258630	−0.846348	−0.306387	0.083*	
C51	−0.2539 (5)	−0.7089 (4)	−0.2306 (4)	0.0693 (16)	
H51	−0.334433	−0.726301	−0.241841	0.083*	
C52	−0.1818 (4)	−0.6112 (4)	−0.1761 (4)	0.0618 (14)	
H52	−0.212778	−0.562268	−0.149741	0.074*	
C53	0.4027 (7)	0.2640 (6)	0.6845 (6)	0.110 (3)	
H53A	0.407802	0.266389	0.625938	0.132*	
H53B	0.433915	0.210544	0.698022	0.132*	

### Atomic displacement parameters (Å^2^)

**Table:**

	*U*^11^	*U*^22^	*U*^33^	*U*^12^	*U*^13^	*U*^23^
Cu1	0.0202 (2)	0.0321 (3)	0.0350 (3)	0.00681 (18)	0.00683 (18)	0.0143 (2)
Cu2	0.0248 (2)	0.0339 (3)	0.0316 (3)	0.01083 (19)	0.01190 (19)	0.0109 (2)
Cu3	0.0261 (2)	0.0339 (3)	0.0293 (2)	0.00812 (19)	0.01021 (19)	0.0118 (2)
Cu4	0.0252 (2)	0.0304 (3)	0.0342 (3)	0.00260 (19)	0.00720 (19)	0.0040 (2)
Cl1	0.0310 (5)	0.0498 (6)	0.0324 (5)	0.0151 (4)	0.0113 (4)	0.0162 (4)
Cl2	0.0222 (4)	0.0430 (6)	0.0423 (5)	0.0042 (4)	0.0066 (4)	0.0219 (5)
Cl3	0.0322 (5)	0.0288 (5)	0.0520 (6)	0.0061 (4)	0.0044 (4)	0.0128 (4)
Cl4	0.0336 (5)	0.0310 (5)	0.0421 (5)	0.0102 (4)	0.0185 (4)	0.0127 (4)
Cl5	0.0220 (4)	0.0426 (6)	0.0341 (5)	0.0057 (4)	0.0083 (4)	0.0064 (4)
Cl6	0.0344 (5)	0.0402 (5)	0.0290 (5)	0.0087 (4)	0.0082 (4)	0.0075 (4)
Cl7	0.138 (3)	0.145 (3)	0.374 (6)	0.052 (2)	0.100 (3)	−0.027 (3)
Cl8	0.1206 (19)	0.152 (2)	0.165 (2)	0.0463 (17)	0.0331 (17)	0.090 (2)
O	0.0205 (12)	0.0264 (13)	0.0275 (13)	0.0051 (10)	0.0082 (10)	0.0064 (11)
N1	0.0239 (16)	0.0360 (18)	0.0373 (18)	0.0072 (13)	0.0087 (14)	0.0158 (15)
N2	0.0283 (16)	0.0366 (19)	0.0360 (18)	0.0131 (14)	0.0154 (14)	0.0134 (15)
N3	0.0310 (17)	0.0411 (19)	0.0314 (17)	0.0152 (15)	0.0149 (14)	0.0164 (15)
N4	0.0333 (18)	0.0343 (19)	0.0374 (19)	0.0037 (15)	0.0090 (15)	0.0049 (15)
C1	0.028 (2)	0.046 (2)	0.048 (2)	0.0095 (18)	0.0134 (18)	0.027 (2)
C2	0.0221 (18)	0.047 (2)	0.047 (2)	0.0079 (17)	0.0087 (17)	0.022 (2)
C3	0.0273 (19)	0.041 (2)	0.038 (2)	0.0128 (17)	0.0101 (17)	0.0141 (19)
C4	0.030 (2)	0.042 (2)	0.051 (3)	0.0070 (18)	0.0108 (19)	0.027 (2)
C5	0.027 (2)	0.048 (3)	0.050 (3)	0.0101 (18)	0.0107 (18)	0.023 (2)
C6	0.029 (2)	0.046 (2)	0.041 (2)	0.0105 (18)	0.0114 (18)	0.018 (2)
C7	0.0270 (19)	0.041 (2)	0.041 (2)	0.0113 (17)	0.0090 (17)	0.0166 (19)
C8	0.031 (2)	0.037 (2)	0.037 (2)	0.0114 (17)	0.0082 (17)	0.0109 (18)
C9	0.033 (2)	0.052 (3)	0.046 (3)	0.017 (2)	0.0127 (19)	0.022 (2)
C10	0.029 (2)	0.069 (3)	0.053 (3)	0.018 (2)	0.012 (2)	0.024 (3)
C11	0.042 (2)	0.067 (3)	0.041 (3)	0.028 (2)	0.010 (2)	0.020 (2)
C12	0.048 (3)	0.058 (3)	0.048 (3)	0.020 (2)	0.015 (2)	0.027 (2)
C13	0.034 (2)	0.049 (3)	0.047 (3)	0.0119 (19)	0.0114 (19)	0.019 (2)
C14	0.038 (2)	0.032 (2)	0.041 (2)	0.0097 (17)	0.0173 (18)	0.0082 (18)
C15	0.032 (2)	0.039 (2)	0.047 (2)	0.0113 (18)	0.0213 (19)	0.013 (2)
C16	0.034 (2)	0.040 (2)	0.035 (2)	0.0146 (18)	0.0174 (17)	0.0134 (18)
C17	0.040 (2)	0.045 (3)	0.046 (3)	0.008 (2)	0.018 (2)	0.001 (2)
C18	0.031 (2)	0.046 (3)	0.044 (2)	0.0029 (19)	0.0143 (19)	0.002 (2)
C19	0.040 (2)	0.038 (2)	0.033 (2)	0.0127 (19)	0.0169 (18)	0.0079 (18)
C20	0.039 (2)	0.042 (2)	0.033 (2)	0.0140 (19)	0.0139 (18)	0.0113 (19)
C21	0.041 (2)	0.043 (2)	0.034 (2)	0.0215 (19)	0.0198 (18)	0.0143 (19)
C22	0.044 (3)	0.048 (3)	0.045 (3)	0.012 (2)	0.019 (2)	0.001 (2)
C23	0.036 (2)	0.066 (3)	0.053 (3)	0.010 (2)	0.018 (2)	0.007 (3)
C24	0.046 (3)	0.062 (3)	0.049 (3)	0.029 (2)	0.025 (2)	0.022 (2)
C25	0.059 (3)	0.042 (3)	0.047 (3)	0.025 (2)	0.024 (2)	0.015 (2)
C26	0.043 (2)	0.043 (3)	0.047 (3)	0.018 (2)	0.021 (2)	0.015 (2)
C27	0.035 (2)	0.053 (3)	0.043 (2)	0.011 (2)	0.0092 (19)	0.022 (2)
C28	0.041 (2)	0.063 (3)	0.046 (3)	0.017 (2)	0.010 (2)	0.031 (2)
C29	0.045 (2)	0.047 (3)	0.048 (3)	0.022 (2)	0.023 (2)	0.028 (2)
C30	0.038 (2)	0.047 (3)	0.042 (2)	0.0066 (19)	0.0136 (19)	0.020 (2)
C31	0.041 (2)	0.044 (2)	0.033 (2)	0.0097 (19)	0.0103 (18)	0.0158 (19)
C32	0.046 (3)	0.063 (3)	0.051 (3)	0.019 (2)	0.020 (2)	0.031 (3)
C33	0.046 (3)	0.062 (3)	0.049 (3)	0.020 (2)	0.019 (2)	0.032 (2)
C34	0.047 (3)	0.048 (3)	0.048 (3)	0.020 (2)	0.021 (2)	0.025 (2)
C35	0.039 (2)	0.047 (3)	0.041 (2)	0.012 (2)	0.0096 (19)	0.018 (2)
C36	0.046 (2)	0.052 (3)	0.043 (2)	0.022 (2)	0.019 (2)	0.025 (2)
C37	0.066 (3)	0.045 (3)	0.056 (3)	0.026 (2)	0.024 (2)	0.030 (2)
C38	0.101 (5)	0.048 (3)	0.054 (3)	0.035 (3)	0.024 (3)	0.019 (3)
C39	0.085 (4)	0.059 (3)	0.048 (3)	0.037 (3)	0.033 (3)	0.027 (3)
C40	0.037 (2)	0.050 (3)	0.060 (3)	0.004 (2)	0.015 (2)	−0.001 (2)
C41	0.049 (3)	0.048 (3)	0.065 (3)	0.009 (2)	0.021 (3)	−0.004 (3)
C42	0.040 (2)	0.034 (2)	0.035 (2)	0.0028 (18)	0.0021 (18)	0.0058 (18)
C43	0.033 (2)	0.041 (3)	0.053 (3)	0.0030 (19)	0.005 (2)	0.006 (2)
C44	0.038 (2)	0.038 (2)	0.049 (3)	0.0090 (19)	0.007 (2)	0.004 (2)
C45	0.048 (3)	0.042 (3)	0.041 (2)	0.007 (2)	0.010 (2)	0.010 (2)
C46	0.049 (3)	0.039 (3)	0.041 (2)	0.006 (2)	0.009 (2)	0.012 (2)
C47	0.048 (3)	0.037 (2)	0.041 (2)	0.000 (2)	0.007 (2)	0.012 (2)
C48	0.058 (3)	0.049 (3)	0.055 (3)	0.001 (2)	0.021 (3)	0.006 (2)
C49	0.073 (4)	0.052 (3)	0.066 (4)	0.002 (3)	0.028 (3)	−0.003 (3)
C50	0.080 (4)	0.048 (3)	0.053 (3)	−0.002 (3)	0.015 (3)	0.005 (3)
C51	0.048 (3)	0.054 (3)	0.080 (4)	−0.004 (3)	−0.001 (3)	0.024 (3)
C52	0.051 (3)	0.046 (3)	0.073 (4)	0.008 (2)	0.006 (3)	0.020 (3)
C53	0.118 (7)	0.096 (6)	0.127 (7)	0.041 (5)	0.052 (6)	0.039 (5)

### Geometric parameters (Å, º)

**Table:**

Cu1—O	1.904 (2)	C18—H18	0.9500
Cu1—N1	1.976 (3)	C19—C20	1.192 (6)
Cu1—Cl2	2.3581 (10)	C20—C21	1.441 (6)
Cu1—Cl3	2.4098 (11)	C21—C22	1.388 (6)
Cu1—Cl1	2.5036 (11)	C21—C26	1.392 (6)
Cu2—O	1.910 (2)	C22—C23	1.376 (6)
Cu2—N2	1.986 (3)	C22—H22	0.9500
Cu2—Cl1	2.3586 (10)	C23—C24	1.373 (7)
Cu2—Cl4	2.3918 (10)	C23—H23	0.9500
Cu2—Cl5	2.4874 (10)	C24—C25	1.376 (7)
Cu3—O	1.895 (2)	C24—H24	0.9500
Cu3—N3	1.972 (3)	C25—C26	1.401 (6)
Cu3—Cl6	2.3586 (11)	C25—H25	0.9500
Cu3—Cl4	2.4316 (11)	C26—H26	0.9500
Cu3—Cl2	2.4602 (10)	C27—C28	1.371 (6)
Cu4—O	1.911 (2)	C27—H27	0.9500
Cu4—N4	1.982 (3)	C28—C29	1.403 (6)
Cu4—Cl5	2.3666 (11)	C28—H28	0.9500
Cu4—Cl6	2.3987 (11)	C29—C30	1.390 (6)
Cu4—Cl3	2.4101 (11)	C29—C32	1.441 (6)
Cl7—C53	1.727 (9)	C30—C31	1.382 (6)
Cl8—C53	1.706 (9)	C30—H30	0.9500
N1—C1	1.331 (5)	C31—H31	0.9500
N1—C5	1.347 (5)	C32—C33	1.185 (6)
N2—C18	1.333 (5)	C33—C34	1.431 (6)
N2—C14	1.338 (5)	C34—C39	1.384 (7)
N3—C31	1.336 (5)	C34—C35	1.391 (6)
N3—C27	1.344 (5)	C35—C36	1.379 (6)
N4—C40	1.312 (6)	C35—H35	0.9500
N4—C44	1.330 (5)	C36—C37	1.365 (7)
C1—C2	1.377 (6)	C36—H36	0.9500
C1—H1	0.9500	C37—C38	1.374 (7)
C2—C3	1.383 (6)	C37—H37	0.9500
C2—H2	0.9500	C38—C39	1.394 (7)
C3—C4	1.398 (5)	C38—H38	0.9500
C3—C6	1.442 (6)	C39—H39	0.9500
C4—C5	1.368 (6)	C40—C41	1.387 (7)
C4—H4	0.9500	C40—H40	0.9500
C5—H5	0.9500	C41—C42	1.392 (6)
C6—C7	1.192 (6)	C41—H41	0.9500
C7—C8	1.443 (5)	C42—C43	1.371 (7)
C8—C13	1.386 (6)	C42—C45	1.427 (6)
C8—C9	1.395 (6)	C43—C44	1.378 (6)
C9—C10	1.385 (6)	C43—H43	0.9500
C9—H9	0.9500	C44—H44	0.9500
C10—C11	1.366 (7)	C45—C46	1.203 (6)
C10—H10	0.9500	C46—C47	1.436 (6)
C11—C12	1.382 (7)	C47—C48	1.375 (7)
C11—H11	0.9500	C47—C52	1.382 (7)
C12—C13	1.369 (6)	C48—C49	1.368 (7)
C12—H12	0.9500	C48—H48	0.9500
C13—H13	0.9500	C49—C50	1.369 (8)
C14—C15	1.369 (6)	C49—H49	0.9500
C14—H14	0.9500	C50—C51	1.377 (8)
C15—C16	1.379 (6)	C50—H50	0.9500
C15—H15	0.9500	C51—C52	1.394 (7)
C16—C17	1.395 (6)	C51—H51	0.9500
C16—C19	1.440 (6)	C52—H52	0.9500
C17—C18	1.378 (6)	C53—H53A	0.9900
C17—H17	0.9500	C53—H53B	0.9900
			
O—Cu1—N1	176.66 (12)	C15—C16—C17	116.8 (4)
O—Cu1—Cl2	85.93 (7)	C15—C16—C19	120.1 (4)
N1—Cu1—Cl2	97.34 (9)	C17—C16—C19	123.2 (4)
O—Cu1—Cl3	84.70 (8)	C18—C17—C16	119.4 (4)
N1—Cu1—Cl3	92.56 (10)	C18—C17—H17	120.3
Cl2—Cu1—Cl3	132.72 (4)	C16—C17—H17	120.3
O—Cu1—Cl1	83.27 (8)	N2—C18—C17	123.4 (4)
N1—Cu1—Cl1	95.83 (10)	N2—C18—H18	118.3
Cl2—Cu1—Cl1	116.16 (4)	C17—C18—H18	118.3
Cl3—Cu1—Cl1	108.55 (4)	C20—C19—C16	174.1 (5)
O—Cu2—N2	176.97 (12)	C19—C20—C21	178.3 (5)
O—Cu2—Cl1	87.21 (8)	C22—C21—C26	120.2 (4)
N2—Cu2—Cl1	95.20 (10)	C22—C21—C20	119.6 (4)
O—Cu2—Cl4	85.66 (8)	C26—C21—C20	120.2 (4)
N2—Cu2—Cl4	94.47 (10)	C23—C22—C21	120.1 (4)
Cl1—Cu2—Cl4	124.17 (4)	C23—C22—H22	119.9
O—Cu2—Cl5	81.79 (7)	C21—C22—H22	119.9
N2—Cu2—Cl5	95.42 (10)	C24—C23—C22	120.2 (4)
Cl1—Cu2—Cl5	118.83 (4)	C24—C23—H23	119.9
Cl4—Cu2—Cl5	114.72 (4)	C22—C23—H23	119.9
O—Cu3—N3	176.99 (12)	C23—C24—C25	120.6 (4)
O—Cu3—Cl6	86.45 (8)	C23—C24—H24	119.7
N3—Cu3—Cl6	95.31 (10)	C25—C24—H24	119.7
O—Cu3—Cl4	84.86 (8)	C24—C25—C26	120.2 (4)
N3—Cu3—Cl4	92.14 (10)	C24—C25—H25	119.9
Cl6—Cu3—Cl4	130.17 (4)	C26—C25—H25	119.9
O—Cu3—Cl2	83.24 (7)	C21—C26—C25	118.7 (4)
N3—Cu3—Cl2	97.90 (9)	C21—C26—H26	120.6
Cl6—Cu3—Cl2	120.96 (4)	C25—C26—H26	120.6
Cl4—Cu3—Cl2	106.52 (4)	N3—C27—C28	122.8 (4)
O—Cu4—N4	177.76 (12)	N3—C27—H27	118.6
O—Cu4—Cl5	85.09 (8)	C28—C27—H27	118.6
N4—Cu4—Cl5	96.99 (11)	C27—C28—C29	119.3 (4)
O—Cu4—Cl6	84.95 (8)	C27—C28—H28	120.3
N4—Cu4—Cl6	94.70 (11)	C29—C28—H28	120.3
Cl5—Cu4—Cl6	119.74 (4)	C30—C29—C28	117.8 (4)
O—Cu4—Cl3	84.53 (8)	C30—C29—C32	120.4 (4)
N4—Cu4—Cl3	93.68 (11)	C28—C29—C32	121.8 (4)
Cl5—Cu4—Cl3	120.61 (4)	C31—C30—C29	118.9 (4)
Cl6—Cu4—Cl3	117.27 (4)	C31—C30—H30	120.5
Cu2—Cl1—Cu1	79.49 (3)	C29—C30—H30	120.5
Cu1—Cl2—Cu3	80.10 (3)	N3—C31—C30	123.2 (4)
Cu1—Cl3—Cu4	80.64 (3)	N3—C31—H31	118.4
Cu2—Cl4—Cu3	80.09 (3)	C30—C31—H31	118.4
Cu4—Cl5—Cu2	81.16 (3)	C33—C32—C29	178.5 (6)
Cu3—Cl6—Cu4	80.40 (3)	C32—C33—C34	176.9 (6)
Cu3—O—Cu1	109.47 (11)	C39—C34—C35	118.9 (4)
Cu3—O—Cu2	109.29 (12)	C39—C34—C33	119.5 (4)
Cu1—O—Cu2	109.31 (12)	C35—C34—C33	121.6 (4)
Cu3—O—Cu4	107.57 (12)	C36—C35—C34	120.6 (4)
Cu1—O—Cu4	109.67 (12)	C36—C35—H35	119.7
Cu2—O—Cu4	111.50 (11)	C34—C35—H35	119.7
C1—N1—C5	116.9 (3)	C37—C36—C35	119.7 (4)
C1—N1—Cu1	124.6 (3)	C37—C36—H36	120.1
C5—N1—Cu1	118.4 (3)	C35—C36—H36	120.1
C18—N2—C14	116.9 (3)	C36—C37—C38	121.1 (4)
C18—N2—Cu2	122.2 (3)	C36—C37—H37	119.4
C14—N2—Cu2	120.9 (3)	C38—C37—H37	119.4
C31—N3—C27	118.0 (4)	C37—C38—C39	119.3 (5)
C31—N3—Cu3	119.7 (3)	C37—C38—H38	120.3
C27—N3—Cu3	121.9 (3)	C39—C38—H38	120.3
C40—N4—C44	117.1 (4)	C34—C39—C38	120.3 (5)
C40—N4—Cu4	118.9 (3)	C34—C39—H39	119.9
C44—N4—Cu4	123.8 (3)	C38—C39—H39	119.9
N1—C1—C2	123.7 (4)	N4—C40—C41	123.4 (4)
N1—C1—H1	118.2	N4—C40—H40	118.3
C2—C1—H1	118.2	C41—C40—H40	118.3
C1—C2—C3	119.4 (4)	C40—C41—C42	119.3 (5)
C1—C2—H2	120.3	C40—C41—H41	120.3
C3—C2—H2	120.3	C42—C41—H41	120.3
C2—C3—C4	117.3 (4)	C43—C42—C41	116.6 (4)
C2—C3—C6	121.7 (4)	C43—C42—C45	120.9 (4)
C4—C3—C6	121.0 (4)	C41—C42—C45	122.5 (4)
C5—C4—C3	119.4 (4)	C42—C43—C44	119.9 (4)
C5—C4—H4	120.3	C42—C43—H43	120.0
C3—C4—H4	120.3	C44—C43—H43	120.0
N1—C5—C4	123.3 (4)	N4—C44—C43	123.3 (4)
N1—C5—H5	118.3	N4—C44—H44	118.3
C4—C5—H5	118.3	C43—C44—H44	118.3
C7—C6—C3	178.0 (4)	C46—C45—C42	176.3 (5)
C6—C7—C8	178.5 (4)	C45—C46—C47	179.1 (6)
C13—C8—C9	119.1 (4)	C48—C47—C52	119.0 (4)
C13—C8—C7	120.8 (4)	C48—C47—C46	120.9 (5)
C9—C8—C7	120.1 (4)	C52—C47—C46	120.1 (5)
C10—C9—C8	119.7 (4)	C49—C48—C47	121.5 (5)
C10—C9—H9	120.1	C49—C48—H48	119.3
C8—C9—H9	120.1	C47—C48—H48	119.3
C11—C10—C9	120.4 (4)	C48—C49—C50	120.0 (6)
C11—C10—H10	119.8	C48—C49—H49	120.0
C9—C10—H10	119.8	C50—C49—H49	120.0
C10—C11—C12	119.9 (4)	C49—C50—C51	119.6 (5)
C10—C11—H11	120.1	C49—C50—H50	120.2
C12—C11—H11	120.1	C51—C50—H50	120.2
C13—C12—C11	120.5 (4)	C50—C51—C52	120.4 (5)
C13—C12—H12	119.7	C50—C51—H51	119.8
C11—C12—H12	119.7	C52—C51—H51	119.8
C12—C13—C8	120.3 (4)	C47—C52—C51	119.5 (5)
C12—C13—H13	119.9	C47—C52—H52	120.2
C8—C13—H13	119.9	C51—C52—H52	120.2
N2—C14—C15	123.2 (4)	Cl8—C53—Cl7	113.3 (5)
N2—C14—H14	118.4	Cl8—C53—H53A	108.9
C15—C14—H14	118.4	Cl7—C53—H53A	108.9
C14—C15—C16	120.3 (4)	Cl8—C53—H53B	108.9
C14—C15—H15	119.9	Cl7—C53—H53B	108.9
C16—C15—H15	119.9	H53A—C53—H53B	107.7
			
Cl6—Cu3—O—Cu1	112.21 (11)	C23—C24—C25—C26	−0.9 (7)
Cl4—Cu3—O—Cu1	−116.90 (11)	C22—C21—C26—C25	−1.5 (6)
Cl2—Cu3—O—Cu1	−9.55 (10)	C20—C21—C26—C25	179.1 (4)
Cl6—Cu3—O—Cu2	−128.10 (10)	C24—C25—C26—C21	1.5 (7)
Cl4—Cu3—O—Cu2	2.78 (9)	C31—N3—C27—C28	−0.3 (7)
Cl2—Cu3—O—Cu2	110.14 (10)	Cu3—N3—C27—C28	172.6 (4)
Cl6—Cu3—O—Cu4	−6.90 (10)	N3—C27—C28—C29	1.1 (7)
Cl4—Cu3—O—Cu4	123.99 (10)	C27—C28—C29—C30	−2.0 (7)
Cl2—Cu3—O—Cu4	−128.66 (11)	C27—C28—C29—C32	−179.5 (4)
C5—N1—C1—C2	−0.8 (7)	C28—C29—C30—C31	2.2 (7)
Cu1—N1—C1—C2	−177.7 (3)	C32—C29—C30—C31	179.7 (4)
N1—C1—C2—C3	0.1 (7)	C27—N3—C31—C30	0.5 (7)
C1—C2—C3—C4	0.6 (6)	Cu3—N3—C31—C30	−172.5 (3)
C1—C2—C3—C6	−178.1 (4)	C29—C30—C31—N3	−1.5 (7)
C2—C3—C4—C5	−0.6 (7)	C39—C34—C35—C36	1.3 (7)
C6—C3—C4—C5	178.1 (4)	C33—C34—C35—C36	179.9 (4)
C1—N1—C5—C4	0.8 (7)	C34—C35—C36—C37	−0.7 (7)
Cu1—N1—C5—C4	177.9 (4)	C35—C36—C37—C38	0.0 (7)
C3—C4—C5—N1	−0.1 (7)	C36—C37—C38—C39	0.1 (8)
C13—C8—C9—C10	−1.7 (7)	C35—C34—C39—C38	−1.2 (7)
C7—C8—C9—C10	177.2 (4)	C33—C34—C39—C38	−179.9 (5)
C8—C9—C10—C11	1.6 (7)	C37—C38—C39—C34	0.5 (8)
C9—C10—C11—C12	−0.7 (8)	C44—N4—C40—C41	−2.9 (8)
C10—C11—C12—C13	−0.2 (8)	Cu4—N4—C40—C41	173.1 (4)
C11—C12—C13—C8	0.1 (7)	N4—C40—C41—C42	−1.5 (9)
C9—C8—C13—C12	0.9 (7)	C40—C41—C42—C43	5.1 (8)
C7—C8—C13—C12	−178.0 (4)	C40—C41—C42—C45	−176.7 (5)
C18—N2—C14—C15	0.3 (6)	C41—C42—C43—C44	−4.3 (7)
Cu2—N2—C14—C15	−177.0 (3)	C45—C42—C43—C44	177.4 (4)
N2—C14—C15—C16	0.2 (7)	C40—N4—C44—C43	3.8 (7)
C14—C15—C16—C17	−0.6 (6)	Cu4—N4—C44—C43	−172.0 (4)
C14—C15—C16—C19	179.2 (4)	C42—C43—C44—N4	−0.1 (8)
C15—C16—C17—C18	0.4 (7)	C52—C47—C48—C49	1.0 (8)
C19—C16—C17—C18	−179.4 (4)	C46—C47—C48—C49	−179.6 (5)
C14—N2—C18—C17	−0.5 (7)	C47—C48—C49—C50	1.3 (9)
Cu2—N2—C18—C17	176.7 (4)	C48—C49—C50—C51	−2.5 (9)
C16—C17—C18—N2	0.1 (7)	C49—C50—C51—C52	1.6 (9)
C26—C21—C22—C23	0.9 (7)	C48—C47—C52—C51	−2.0 (8)
C20—C21—C22—C23	−179.7 (4)	C46—C47—C52—C51	178.6 (5)
C21—C22—C23—C24	−0.3 (8)	C50—C51—C52—C47	0.7 (9)
C22—C23—C24—C25	0.3 (8)		

### Hydrogen-bond geometry (Å, º)

π is the midpoint of the C19≡C20 triple bond. *Cg*1 and *Cg*2 are the centroids of the N3/C27–C31 and C34–C39 rings, respectively.

**Table:**

*D*—H···*A*	*D*—H	H···*A*	*D*···*A*	*D*—H···*A*
C1—H1···Cl2	0.95	2.64	3.283 (4)	126
C4—H4···Cl5^i^	0.95	2.90	3.697 (4)	142
C14—H14···Cl5	0.95	2.52	3.211 (4)	130
C17—H17···Cl4^ii^	0.95	2.80	3.609 (5)	144
C27—H27···Cl6	0.95	2.74	3.297 (5)	118
C31—H31···Cl2	0.95	2.71	3.345 (4)	125
C44—H44···Cl5	0.95	2.61	3.257 (5)	126
C53—H53*A*···Cl1	0.99	2.97	3.808 (9)	143
C53—H53*B*···Cl3^i^	0.99	2.79	3.771 (8)	172
C31—H31···π	0.95	2.84	3.600 (3)	135
C41—H41···*Cg*1^iii^	0.95	2.78	3.705 (6)	165
C43—H43···*Cg*2	0.95	2.73	3.452 (2)	150 (1)

Symmetry codes: (i) −*x*+1, −*y*, −*z*+1; (ii) −*x*+1, −*y*+1, −*z*+1; (iii) −*x*+1, −*y*, −*z*.
